# High-resolution adaptive optics-trans-scleral flood illumination (AO-TFI) imaging of retinal pigment epithelium (RPE) in central serous chorioretinopathy (CSCR)

**DOI:** 10.1038/s41598-024-64524-4

**Published:** 2024-06-13

**Authors:** Vishal Govindahari, Rémy Dornier, Sohrab Ferdowsi, Christophe Moser, Irmela Mantel, Francine Behar-Cohen, Laura Kowalczuk

**Affiliations:** 1 Department of Retina, Pushpagiri Eye Institute, Hyderabad, 500026 India; 2 INSERM UMRS 1138 From Physiopathology of Ocular Diseases to Clinical Developments, Centre de Recherche des Cordeliers, Université Pierre et Marie Curie - Paris 6, 75006 Paris, France; 3 Laboratory of Applied Photonic Devices (LAPD), School of Engineering, École Polytechnique Fédérale de Lausanne (EPFL), CH-1015 Lausanne, Switzerland; 4 Earlysight SA, CH-1202 Geneva, Switzerland; 5 Jules-Gonin Eye Hospital, Fondation Asile des Aveugles, CH-1004 Lausanne, Switzerland; 6 Faculty of Biology and Medicine, University of Lausanne, CH-1005 Lausanne, Switzerland; 7 Assistance Publique - Hôpitaux de Paris, Ophtalmopôle, Cochin Hospital, 75014 Paris, France; 8 Université Paris Cité, 75006 Paris, France; 9 Hôpital Foch, Suresnes, France

**Keywords:** Retinal diseases, Diagnostic markers

## Abstract

This study aims to correlate adaptive optics-transscleral flood illumination (AO-TFI) images of the retinal pigment epithelium (RPE) in central serous chorioretinopathy (CSCR) with standard clinical images and compare cell morphological features with those of healthy eyes. After stitching 125 AO-TFI images acquired in CSCR eyes (including 6 active CSCR, 15 resolved CSCR, and 3 from healthy contralateral), 24 montages were correlated with blue-autofluorescence, infrared and optical coherence tomography images. All 68 AO-TFI images acquired in pathological areas exhibited significant RPE contrast changes. Among the 52 healthy areas in clinical images, AO-TFI revealed a normal RPE mosaic in 62% of the images and an altered RPE pattern in 38% of the images. Morphological features of the RPE cells were quantified in 54 AO-TFI images depicting clinically normal areas (from 12 CSCR eyes). Comparison with data from 149 AO-TFI images acquired in 33 healthy eyes revealed significantly increased morphological heterogeneity. In CSCR, AO-TFI not only enabled high-resolution imaging of outer retinal alterations, but also revealed RPE abnormalities undetectable by all other imaging modalities. Further studies are required to estimate the prognosis value of these abnormalities. Imaging of the RPE using AO-TFI holds great promise for improving our understanding of the CSCR pathogenesis.

## Introduction

Central serous chorioretinopathy (CSCR) stands as the fourth most prevalent retinal diseases^1^. It is characterized by serous detachments of the neurosensory retina often associated with focal detachments of retinal pigment epithelium (RPE)^2^. The pathogenic mechanisms of this complex, multifactorial disease are not fully understood. Advances in multimodal imaging techniques have considerably improved the accuracy of CSCR diagnosis and our understanding of its pathophysiology^3,4^. Within the spectrum of pachychoroid, alterations in the RPE monolayer, referred to as pachychoroid pigment epitheliopathy^5^, may precede the occurrence of RPE barrier leak, and the classification and severity of CSCR is determined by the extent of RPE alterations^6,7^. While numerous studies have focused on imaging and characterizing the choroidal changes in CSCR^4,8,9^, the RPE monolayer, which is crucial for understanding the disease, remains challenging to visualize and has thus not been fully explored.

Spectral-domain optical coherence tomography (OCT) shows various types of pigment epithelial detachment (PED), from serous PED in acute disease to flat, irregular PED in choroidal neovascular membranes associated with CSCR^10^, and degeneration and atrophy of the RPE layer in chronic/complex CSCR^2,3,8^. Besides OCT, clinicians use blue autofluorescence (BAF) and near infrared autofluorescence (NIRAF) to assess the health of the RPE, where lipofuscin and melanin are the main source of signal respectively^3,11–13^. In NIRAF imaging, the signal originates from RPE melanin, and, to some extent, from melanin in the choroidal layers^14^. Fundus pattern descriptions differ according to the disease progression, reflecting changes in the RPE and outer retina^3,15^. However, these conventional imaging techniques do not provide a visual representation of the RPE's structural integrity at the cellular level due to their limitations in lateral resolution.

Adaptive optics (AO) ophthalmoscopy, providing a lateral resolution as fine as 2–3 microns, has been successful in visualizing the photoreceptor mosaic in CSCR^16–20^. AO scanning laser ophthalmoscopy (SLO) images over small field of view (FOV), typically around 1–2°, and flood illumination combined with AO revealed cone mosaic and density changes in resolved CSCR^16–18^, in eyes treated with focal laser^19^, as well as in asymptomatic fellow eyes^20^. However, in these transpupillary systems, the overlying reflective photoreceptors impede RPE imaging^21^. Various AO-based imaging approached have been developed to image the RPE layer. These include AO-OCT^22–24^, combination of AO-SLO with short wavelength AF^25^, dark-field reflectance imaging^26^ or NIRAF^27,28^, and multimodal approaches^29–31^. Despite these improvements, AO-SLO RPE imaging still requires a long acquisition time to accumulate enough frames with a strong enough signal to visualize RPE cells.

Adaptive-optics trans-scleral flood illumination (AO-TFI) is an innovative technology that enables visualization of the RPE monolayer as a dark-field image with minimal interference from the overlying neurosensory retina. This approach relies on oblique illumination of the posterior pole through the sclera using two IR light emitting diodes, coupled with a trans-pupillary AO full-field camera system. AO-TFI provides a 60 µm depth of field and a lateral resolution of 3 µm^32^. Its advantages include a larger FOV (5° × 5°) and quicker acquisition times (< 10 s) compared to AO-SLO RPE imaging. The clinical prototype for AO-TFI used in this study was previously assessed in healthy volunteers, showing the ability to visualize RPE cells and yield reproducible measurements^33^.

The aim of this study was to correlate high-resolution AO-TFI images of the RPE to current imaging techniques for CSCR diagnosis, including BAF and IR fundus images, as well as OCT B-scans. Additionally, the morphological features of RPE cells in healthy appearing areas in CSCR eyes were compared to those of healthy eyes.

## Results

Twenty-four eyes of 14 patients with a clinical diagnosis of CSCR, 11 males and 3 females, with a mean age of 43.4 ± 5.3 years, and 33 eyes from 19 healthy volunteers, 12 males and 7 females, with a mean age of 36.7 ± 12.6 years were included in the analyses. Descriptive statistics for the baseline characteristics of the eyes included in the study are provided in Supplementary Table S1 online.

After screening for the best-contrasted images in each of the predefined imaging areas, 125 AO-TFI images of CSCR patients were included in the correlative analysis. Thirty-three images (26%) were acquired in 6 eyes presenting “active CSCR”, 77 images (62%) in 15 eyes with “resolved CSCR” and 15 images (12%) in 3 healthy contralateral (CL) eyes. The images were initially graded for changes observed in en-face images, which include BAF fundus, IR fundus and AO-TFI images (see Supplementary Table S2 online). Subsequently, mosaics were generated by stitching together AO-TFI images acquired from each eye (see Supplementary Fig. S1 online) and the resulting 24 montages were analyzed using a semi-automated method. This analysis involved the registration of the mosaics with IR fundus images (see Supplementary Fig. S2 online) and the correlation of the warped AO-TFI mosaics with IR fundus and their corresponding OCT B-scans (see Supplementary Fig. S3 online).

### Clinically pathological areas

#### Grade 1: neurosensory detachment

Seven AO-TFI images (6% of the total images) were acquired in areas corresponding to neurosensory detachment (NSD) in 5 out of the 6 active CSCR eyes. Macular (n = 3) and peripheral (n = 2) NSD were documented.

**Figure 1:**
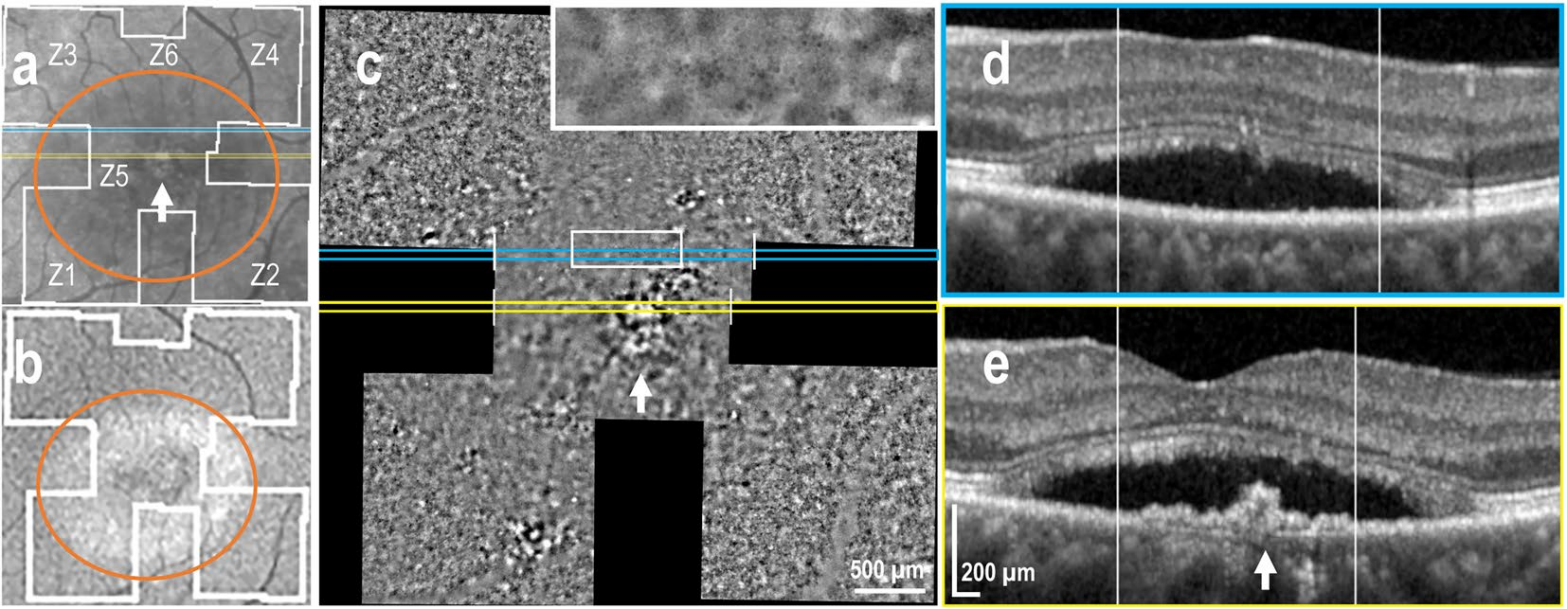
Foveal serous detachment (Left eye, Female, 43 years). (**a**) Infrared fundus image with projection of the AO-TFI mosaic mask (Z5-Z6, Grade 1; Z1 to Z4, Grade 2). The colored lines indicate the locations of the OCT B-scan shown in panels d (blue line) and e (yellow line). (**b**) Blue-autofluorescence fundus image indicating the approximate hyper-reflective areas of NSD (orange circle) and the one imaged with Cellularis (white lines). (**c–e**) Correlation of the AO-TFI mosaic with OCT B-scans showing RPE layer under sub-retinal fluid (**d**) where RPE cells are imaged with AO-TFI (**c**, top blue line) and hyperreflective content (**e**, white arrow) where AO-TFI shows also hyper-reflective content in greater detail (**c**, white arrow) than IR fundus (**a**, white arrow). The complete “IR fundus—AO-TFI—OCT B-scan” correlation is available in the supplementary Movie S2.

Figure 1 illustrates a representative case of macular NSD appearing as hyper-reflective dots on a diffuse hypo-reflective background in IR fundus images (Fig. 1a) and as granular hyper-autofluorescence on BAF images (Fig. 1b). The center of the AO-TFI montage shows contrast changes (Fig. 1c). The central blurred area corresponds to the area of serous detachment in the OCT sections (Fig. 1d and 1e), and the well-defined area with bright components surrounded by a darker edge (Fig. 1c, white arrow) corresponds to an irregular pigment epithelial detachment (PED) in OCT B-scans, also visible on the IR fundus as an ill-defined hyper-reflective area (Fig. 1a and 1e, white arrow). The presence of RPE cells in in the superior region of the blurred area on the AO-TFI montage (Fig. 1c, inset) correlates with an intact RPE layer in the OCT image (Fig. 1d).

**Figure 2:**
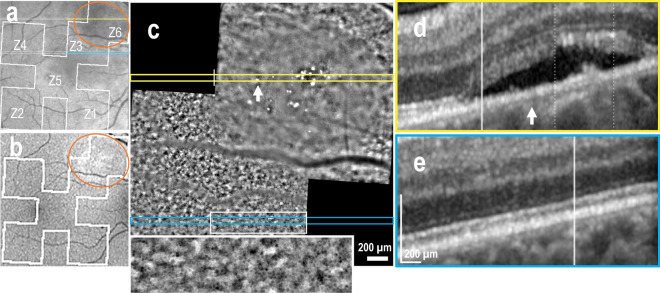
Supero-nasal serous detachment (Right eye, Male, 40 years). (**a**) Infrared fundus image with projection of the AO-TFI mosaic mask (Z6 Grade 1; Z3 Grade 2; Z1, Z2, Z4, Grade 0; Z5 n/a). The colored lines indicate the locations of the OCT B-sections shown in panels d (yellow line) and e (blue line). (**b**) Blue-autofluorescence fundus image indicating the approximate hyper-reflective area of NSD (orange circle) and the one imaged with Cellularis® (white lines). (**c–e**) Correlation of the magnified AO-TFI image with OCT B-scans showing NSD, RPE bump and hyper-reflective foci (**d**) where AO-TFI highlights hyper-reflective dots (**c**, **top yellow line**) and attached retina (**e**) where AO-TFI shows RPE cells mosaic (**c**, **inset**). The complete “IR fundus—AO-TFI—OCT B-scan” correlation is available in the supplementary Movie S3.

In extra-macular NSD, granular and irregular hyper-autofluorescence was observed in BAF images (Fig. 2b), while a darker area in the IR fundus image suggested the presence of subretinal fluid (SRF) (Fig. 2a), which was confirmed by the OCT B-scan (Fig. 2d). The AO-TFI image corresponding to the NSD (Regions Z3 and Z6 in Fig. 2a) reveals an area with poor RPE visualization due to SRF, along with numerous bright dots (Fig. 2c, yellow line) that correlated with the B-scan indicating elongation of photoreceptor outer segments and hyper-reflective foci (Fig. 2d). The AO-TFI image acquired next to the NSD shows RPE cells (Fig. 2c, blue line and inset) that appear aligned on the B-scan (Fig. 2e).

#### Grade 2: altered BAF, IR and AO-TFI images

Grade 2 was observed in 39 AO-TFI images (31% of the total) of eyes with active (12 images) and resolved (27 images) CSCR. Contrast changes in the AO-TFI images were observed in regions considered pathological using conventional en-face imaging.

**Figure 3:**
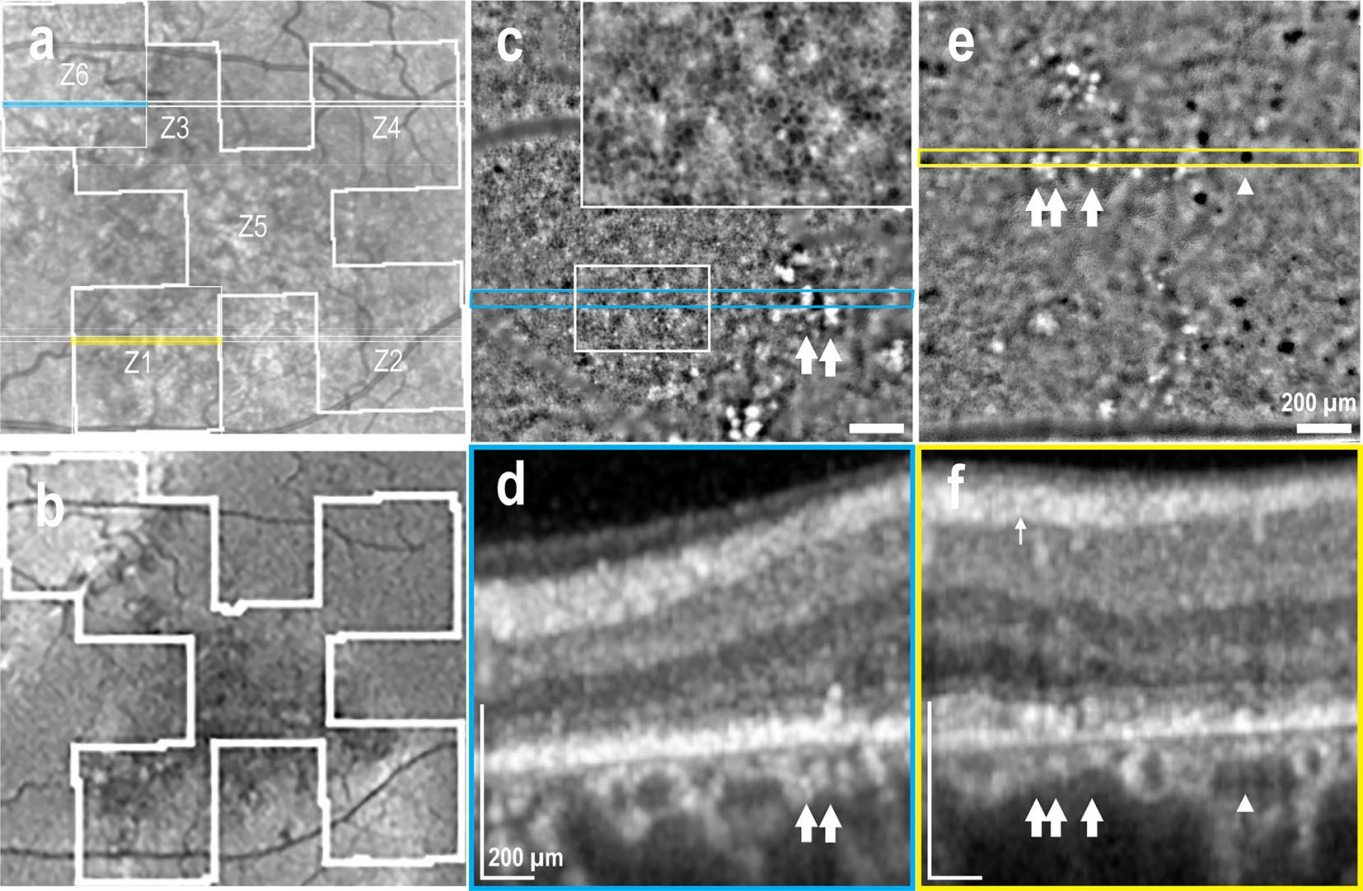
Retinal atrophy in resolved CSCR (Left eye, Male, 47 years). (**a**) Infrared fundus image with projection of the AO-TFI mosaic mask (Z1, Z2, Z5, Z3-Z6: Grade 2; Z4 Grade 3). The colored lines indicate the locations of the OCT B-sections shown in panels d (blue line) and f (yellow line). (**b**) Blue-autofluorescence fundus image indicating the approximate area imaged with Cellularis® (white lines). (**c**, **d**) In outer retinal atrophy, the supero-nasal AO-TFI image shows RPE mosaic (**c**, inset) corresponding to hyper-fluorescence on BAF and loss of RPE digitation on OCT B-scan (**d**). (**e**, **f**) In RPE outer retina atrophy, no RPE cells are visible on the infero-nasal AO-TFI image (**e**) corresponding to hypo-fluorescence on BAF and loss of RPE on OCT B-scan (**f**). Large dark dots are also observed on AO-TFI images (**e**, arrowhead). In both cases, hyperreflective foci on AO-TFI images (**c**, **e**; white arrows) correspond to areas with RPE cell migration on OCT section (**d**, **f**; white arrows). The complete “IR fundus—AO-TFI—OCT B-scan” correlation is available in the supplementary Movie S4.

Two eyes with resolved CSCR presented outer retinal atrophy (ORA) by OCT, with one showing ORA in 2 areas imaged with AO-TFI, while the other eye displayed ORA in one area, and focal RPE and outer retinal atrophy (RORA) in three areas. In this case illustrated in Fig. 3, the ORA region appears predominantly hyper-reflective in the IR fundus image and hyperfluorescent in the BAF image (Region Z6 in Fig. 3a and b), and exhibits loss of RPE digitation by OCT (Fig. 3d). The RPE mosaic is preserved in the AO-TFI image of ORA, showing a network of dark cells surrounded by small bright dots (Fig. 3c, inset). The RORA area appears mostly hypo-reflective with hyper-reflective dots in the IR fundus image and hypo-fluorescent in the BAF image (Regions Z1, Z2 and Z5 in Fig. 3a and b), and presents atrophic RPE by OCT (Fig. 3f). No RPE cells are detected in the AO-TFI image of RORA, demonstrating an atypical pattern with numerous dark dots (Fig. 3e, arrowhead) corresponding to the photoreceptor degeneration observed by OCT. In both cases, clusters of hyper-reflective dots in AO-TFI images (Fig. 3c and e, white arrows) correlate with areas of pigment migration in OCT B-scans (Fig. 3d and f, white arrows).

#### Grade 3: normal BAF images, altered IR and AO-TFI images

Grade 3 was observed in 22 AO-TFI images (18% of the total) of eyes with active (4 images) and resolved (18 images) CSCR.

**Figure 4:**
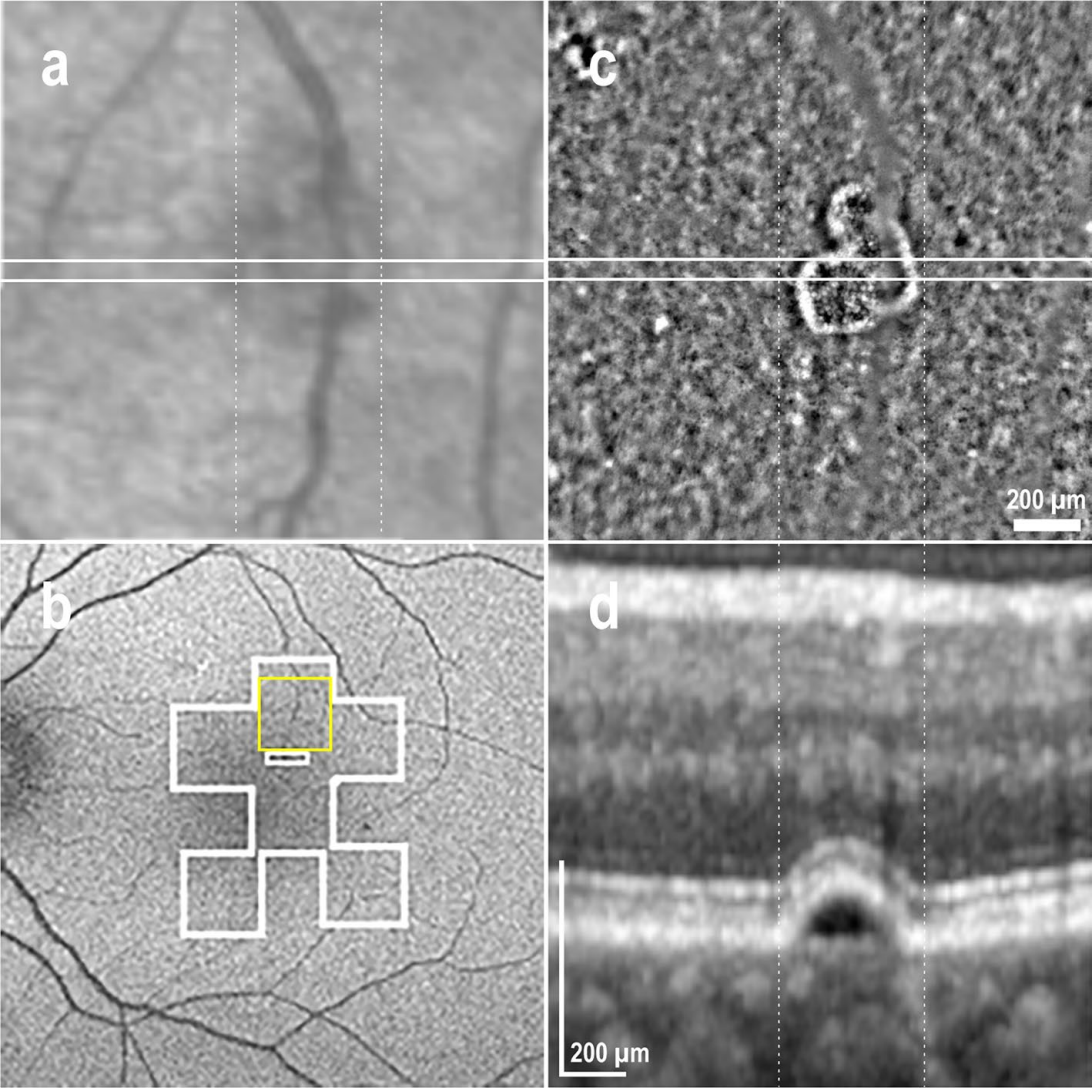
Pigment epithelial detachment (PED) in resolved CSCR (Left eye, Male, 46 years). (**a**) IR fundus area corresponding to the superior AO-TFI image shown in (**c**). The white line indicates the location of the corresponding OCT B-section (**d**). (**b**) Normal blue-autofluorescence fundus image indicating the approximated area imaged using AO-TFI (white lines) and the location of the magnified AO-TFI image (yellow square). (**c**, **d**) Correlation of the AO-TFI image with OCT B-scans showing PED. The vertical dot lines report the width of the distinct area sharply imaged with AO-TFI. The complete “IR fundus—AO-TFI—OCT B-scan” correlation is available in the supplementary Movie S5.

Four AO-TFI images were acquired in areas showing serous PED in the OCT sections of one eye with active CSCR (see supplementary Movie S7 online) and three eyes with resolved CSCR. Only one of these PED was not detectable in the BAF image (Fig. 4b). This persistent localized serous PED visible in the OCT section (Fig. 4d) appears as a hypo-reflective area in the IR fundus image (Fig. 4a). In the AO-TFI image, the PED is depicted as a distinct area composed of small, dense hyper-reflective dots, surrounded by a large bright border (Fig. 4c), which is typical of elevated photoreceptors in a singular pattern.

**Figure 5:**
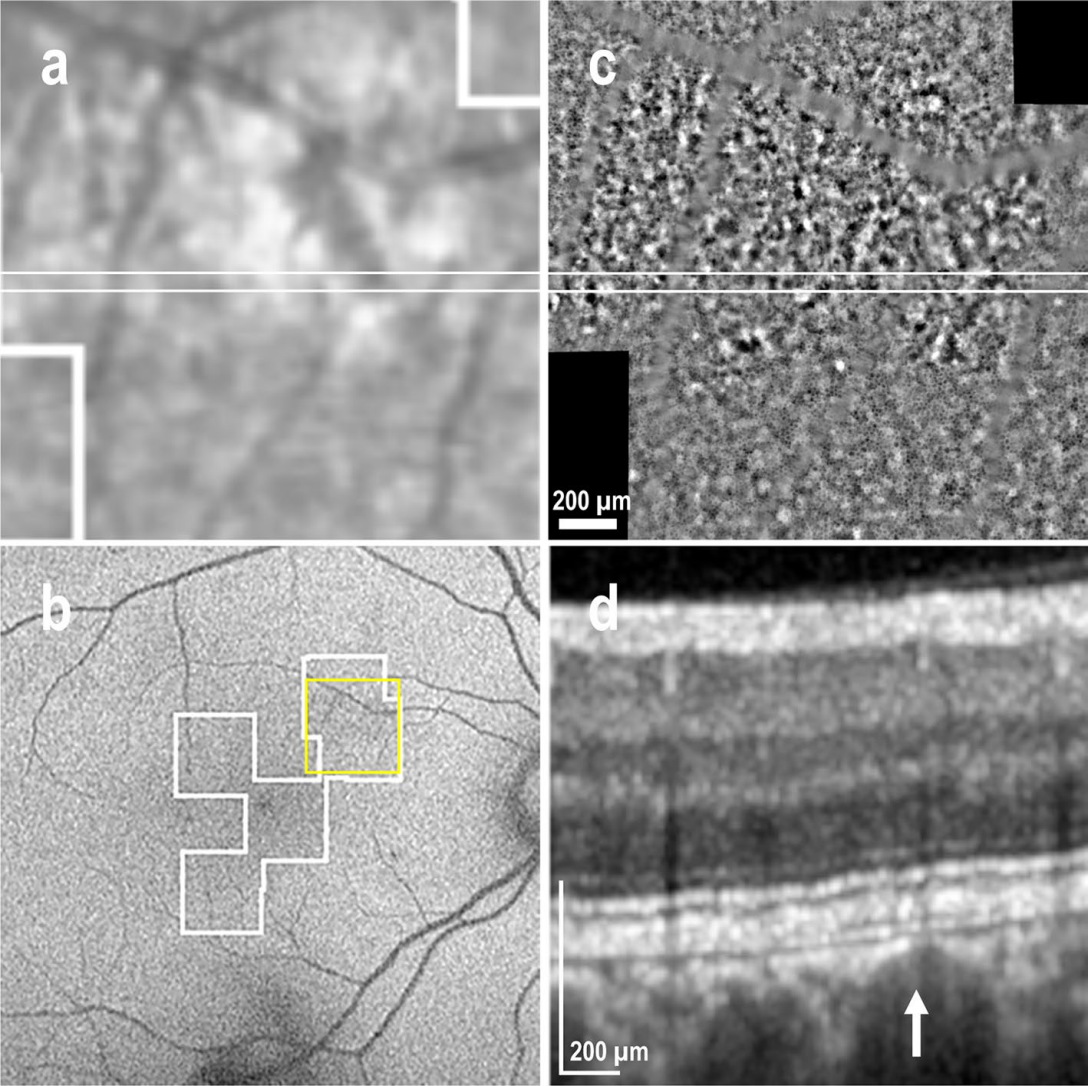
RPE changes on multifocal hyperreflective infrared areas in resolved CSCR (Male, 41 years). (**a**) IR fundus area corresponding to the supero-nasal AO-TFI image (**c**) presenting a large area of abnormal RPE contrast (upper half). The white lines indicate the corresponding OCT B-scan (**d**). (**b**) Normal blue-autofluorescence fundus image indicating the approximated area imaged with Cellularis® (white lines) and the location of the magnified AO-TFI image (yellow square). (**c**, **d**) Correlation of the AO-TFI image showing contrast changes (**c**) where OCT B-scan (**d**) shows irregular alignment of the RPE and effacement of the choriocapillaris (arrow). Lower half of the AO-TFI image shows normal RPE cells.

**Figure 6:**
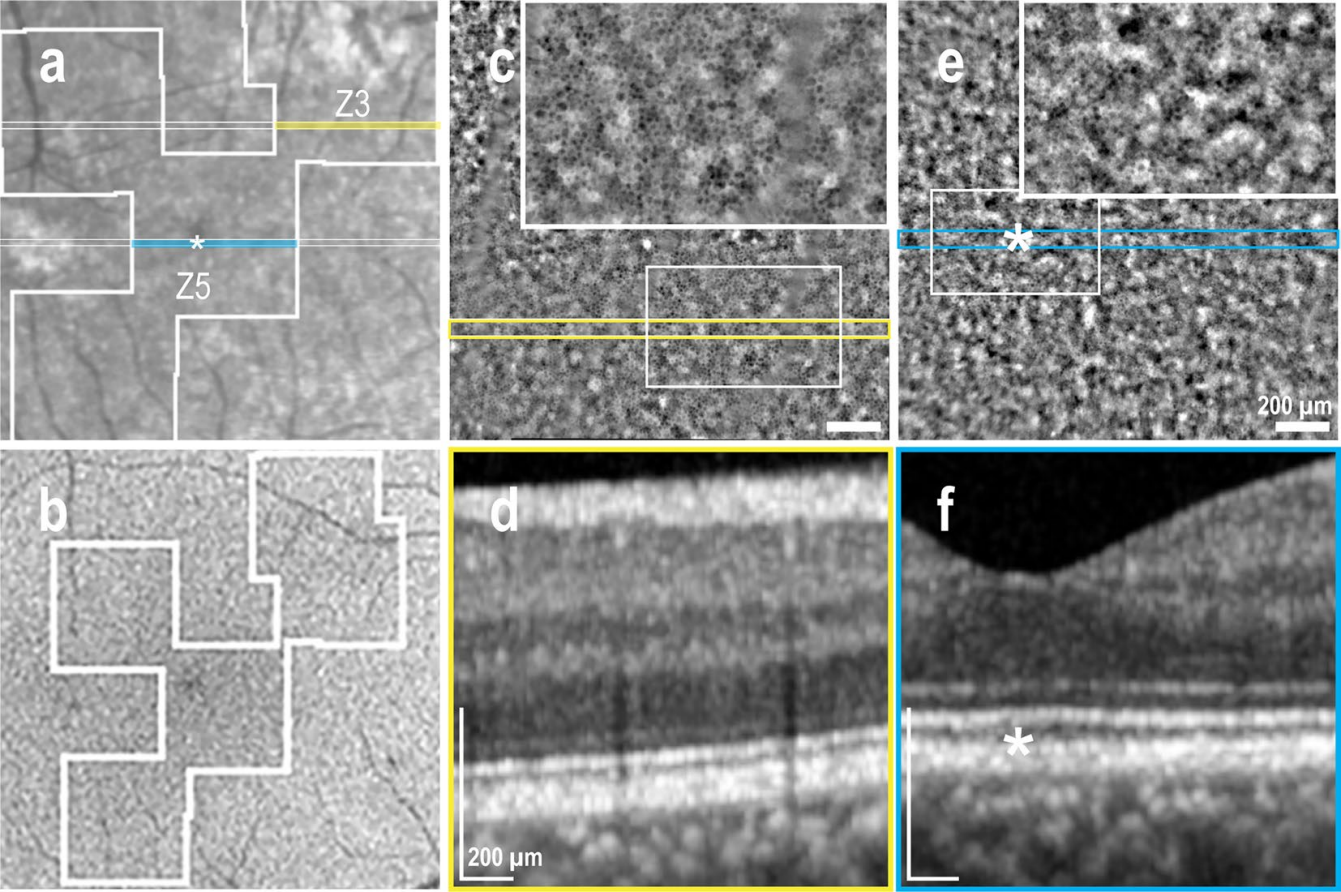
Normal RPE in resolved CSCR (Male, 41 years). (**a**) Infrared fundus image with projection of the mask of the AO-TFI mosaic (Z4, Z3-Z6: Grade 3; Z2, Z5: Grade 0). The colored lines indicate the locations of the OCT B-sections shown in panels d (yellow line) and f (blue line). (**b**) Normal blue-autofluorescence fundus image indicating the approximated area imaged with Cellularis® (white lines). (**c-d**) Correlation of the supero-nasal AO-TFI image (**c**) with the corresponding OCT B-scan (**d**). In the lower half of the AO-TFI image, RPE mosaic is visible (**c**, inset) where the fundus is isoreflective in the infrared image and iso-fluorescent in the blue-autofluorescence image (Z3 in** a** and **b**) and the RPE is above normal choriocapillaris on OCT sections (**d**). (**e**, **f**) Correlation of the magnified foveal AO-TFI image (**e**) with the corresponding OCT B-scan (**f**) showing that healthy RPE cells (**e**, inset) were successfully imaged in the center of the fovea (asterisk). The complete “IR fundus—AO-TFI—OCT B-scan” correlation is available in the supplementary Movie S6.

In another case of resolved CSCR, presenting a normal BAF (Fig. 5b) and multifocal hyper-reflective areas in the IR fundus image (Fig. 5a), the corresponding AO-TFI image (Fig. 5c) reveals a clear contrast change in the RPE monolayer. OCT shows a subtly irregular alignment of the RPE and focal effacement of the choriocapillaris (Fig. 5d, white arrow). The honeycomb pattern in the AO-TFI image corresponds to well-aligned RPE above the normal choriocapillaris in the OCT section (Figs. 6c inset, and 6d).

### Clinically healthy areas

In AO-TFI images, the normal RPE mosaic shows polygonal cells with hypo-reflective centers and bright edges, arranged in a honeycomb pattern. The non-uniform pigmentation of the cells, along with the superimposition of background from adjacent layers, results in a mixed reflectance background within the mosaic (see supplementary Movie S1 online).

Among the 52 AO-TFI images acquired in clinically healthy areas, 32 images showed the normal RPE pattern (62% of images acquired in healthy regions): 5 images in 3 eyes with active CSCR, located away from the region with NSD (see supplementary Movie S3 online), 18 images in 8 resolved CSCR eyes, and 9 images in 2 healthy CL eyes. Healthy foveal RPE cells were successfully imaged in 5 eyes, 3 healthy CL and 2 resolved CSCR (Fig. 6e, inset).

#### Grade 4, normal BAF, and IR images, altered AO-TFI images

Interestingly, in 38% of the images acquired in healthy regions, AO-TFI images showed RPE abnormalities in areas where no changes were observed in BAF or IR fundus images, in all classes of CSCR eyes. Out of the 20 grade 4 images, 5 (25%) exhibited altered mosaic contrast, 12 (60%) showed hypo-reflective dots, and 10 (50%) displayed clusters of hyperreflective dots/foci.

**Figure 7:**
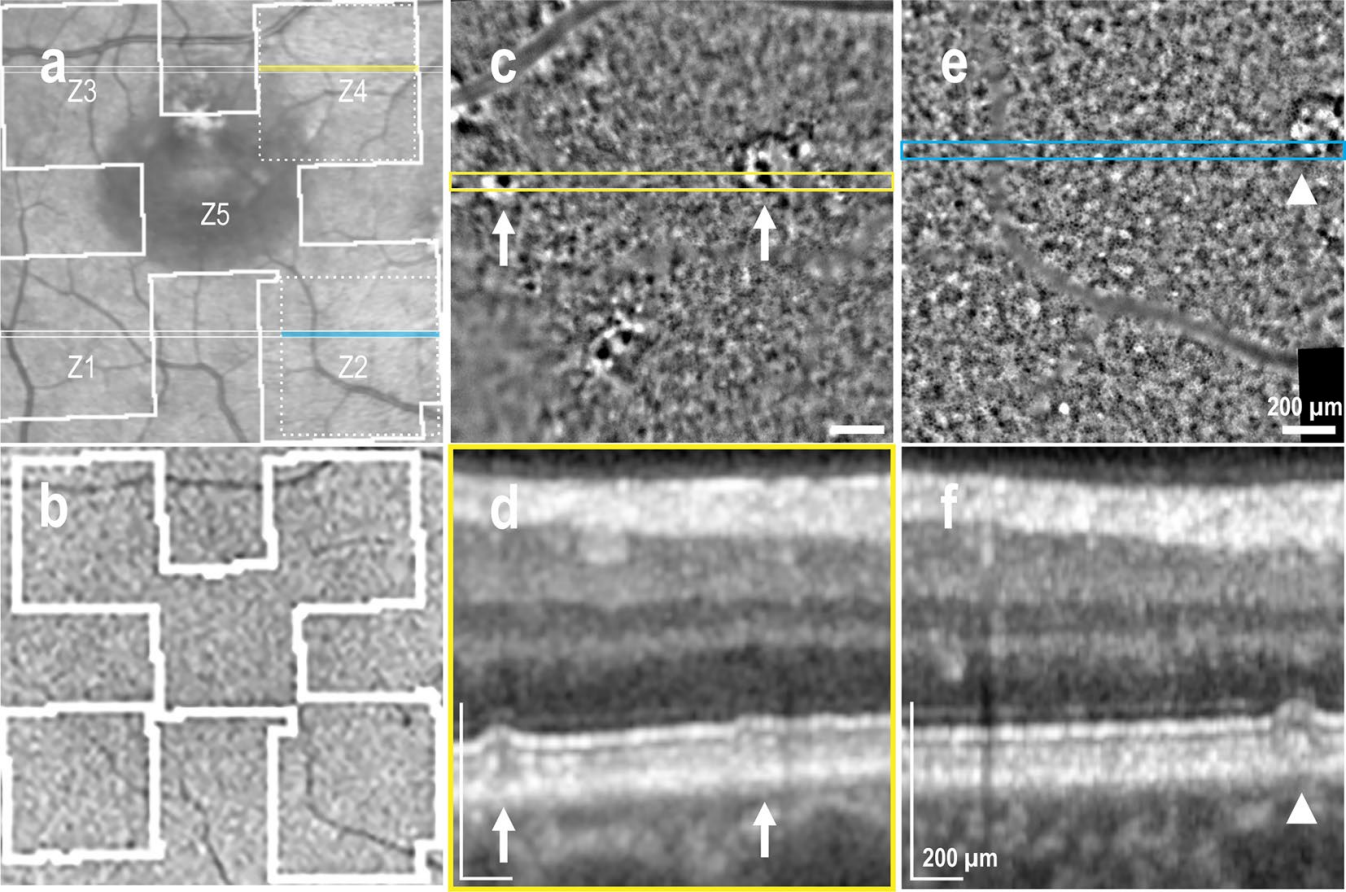
RPE changes on normal BAF and IR fundus areas in active CSCR (Male, 39 years). (**a**) Infrared fundus image with projection of the AO-TFI mosaic mask indicating the AO-TFI images magnified in panels c and e (dot squares). The colored lines indicate the locations of the OCT B-sections shown in panels d (yellow line) and f (blue line). (**b**) Blue-autofluorescence fundus image indicating the approximated area imaged with Cellularis® (white square). (**c-d**) Correlation of the supero-nasal AO-TFI image (**c**) with the corresponding OCT B-scan (**d**). (**e–f**) Correlation of the infero-nasal AO-TFI image (**e**) with the corresponding OCT B-scan (**f**). AO-TFI images show preservation of the RPE cell mosaic and major changes, hypo-reflective areas surrounded by hyperreflective structures (arrows) and hyper-reflective foci (arrowhead) corresponding to loss of alignment of the RPE on OCT sections. The complete “IR fundus – AO-TFI – OCT B-scan” correlation is available in the supplementary Movie S7.

For example, in active CSCR, AO-TFI images acquired in areas around the NSD (Z4 and Z2 in Fig. 7a) reveal preserved mosaic patterns with major contrast changes, such as hypo-reflective areas surrounded by hyper-reflective structures (Fig. 7c, white arrow) and hyper-reflective foci (Fig. 7e, arrowhead). These changes correspond to subtle changes in OCT sections, indicating a slight misalignment between the RPE apical villosities and the outer segments of the photoreceptors (Fig. 7d and f). Small hypo-reflective areas surrounded by hyper-reflective structures are also noted in resolved CSCR, alongside OCT sections indicating slight RPE misalignment with a thin choriocapillaris, or no change (see Supplementary Fig. S4 online).

In summary, in active CSCR, 33 AO-TFI images were acquired in areas with serous detachment (Grade 1). Approximately half of the AO-TFI images were recorded in eyes with active and resolved CSCR, where RPE abnormalities were observed in all three en-face modalities (39 images with grade 2), or only in IR fundus and AO-TFI images (22 images with grade 3). Among the remaining 52 images acquired in clinically healthy areas, AO-TFI revealed a normal RPE mosaic in 62% of these images, but also RPE abnormalities in 38% of the images acquired in healthy regions (20 images, Grade 4).

### Quantitative analysis of RPE cell features in clinically healthy areas

All grades of perifoveal images (Z1 to Z4) from CSCR eyes were included in the comparison of the morphological features of normal RPE mosaics, including hypo-reflective intracellular areas assumed to contain pigments such as melanin and melanolipofuscin granules, with those of healthy controls eyes. Images from areas selected at the physician’s discretion, foveal images, and images from healthy contra-lateral eyes of patients were excluded from this analysis. After selecting images and eyes with matching baseline characteristics (see flow diagram in the Supplementary Fig. S5 online), morphometric measurements were compared between 54 perifoveal images from 12 eyes of 10 CSCR patients and 149 perifoveal images from 33 eyes of 19 healthy volunteers.

**Figure 8:**
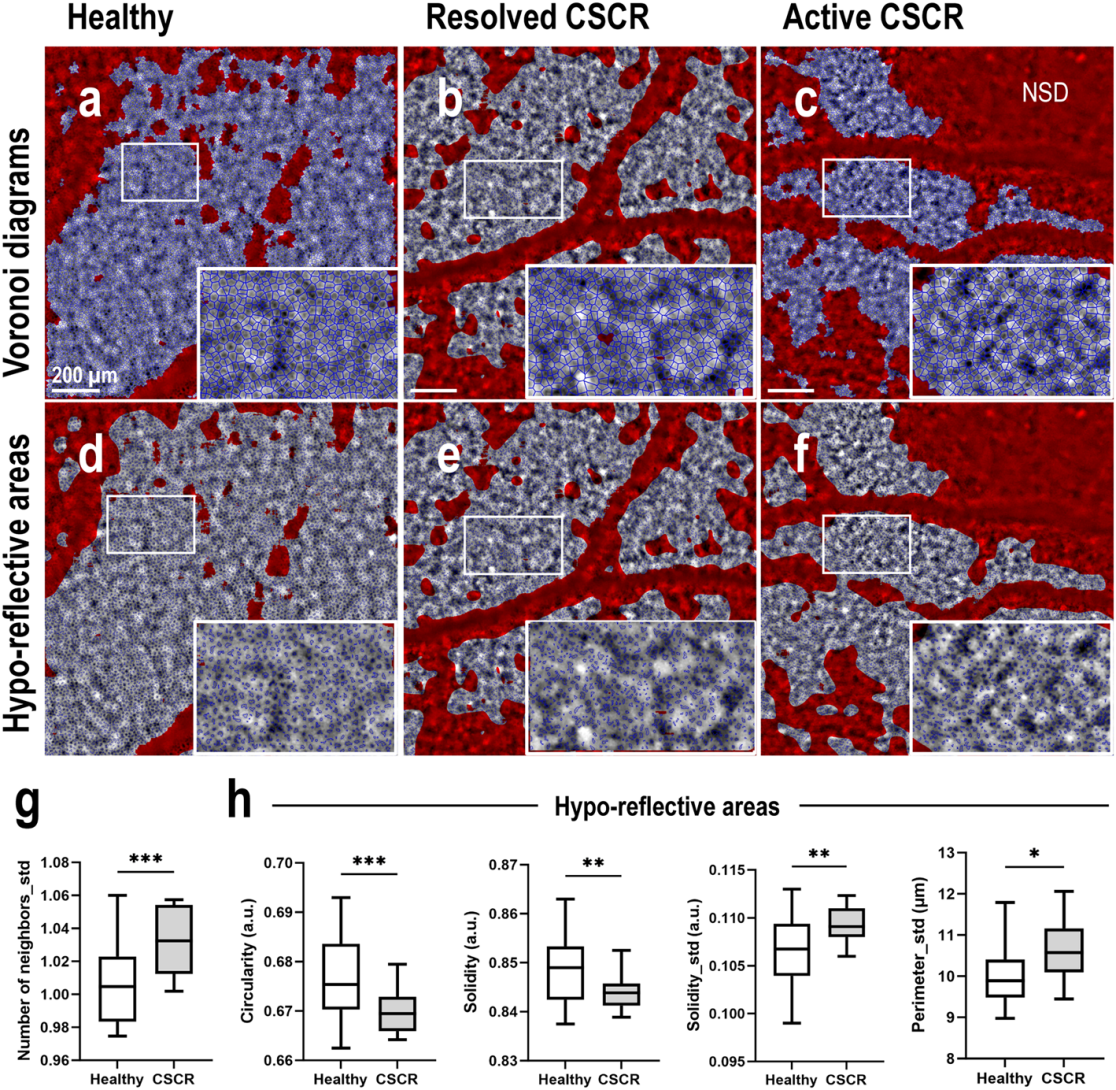
Morphological features in healthy control and CSCR eyes. (**a**–**c**) Visual of tessellation based on Voronoi segmentation in (**a**) healthy CL eye, (**b**) resolved and (**c**) active CSCR. (**d**–**f**) Visual of the hypo-reflective areas segmentation in (**d**) healthy CL, (**e**) resolved and (**f**) active CSCR eyes. (**g**) Comparison of the means of standard deviation of the number of neighboring cells measured on Voronoi diagrams in peri-foveal images from Healthy control and CSCR eyes. (**h**) Comparison of hypo-reflective area morphological features measured after segmentation in peri-foveal images from Healthy control (N = 33) and CSCR (N = 12) eyes.

Figure 8 illustrates the visual results of two types of image analysis: the Voronoi-based tessellation (Fig. 8a–c) and the hypo-reflective area segmentation (Fig. 8d–f), in both healthy and CSCR eyes.

The supplementary Table S3 provides statistics on the morphological features of cell mosaics after tessellation. The comparisons between CSCR and healthy eyes show no difference in density, area (mean and SD), perimeter (mean and SD), diameter (mean and SD) and number of neighboring cells (mean) of the Voronoi diagrams. However, a significant difference is observed between the means of the standard deviations of the number of neighboring cells (*P* < 0.001, Fig. 8g). This suggests that CSCR RPE presents well organized cells, similar to healthy RPE, but with a greater dispersion in the number of neighbors compared to healthy RPE.

The supplementary Table S4 online provides statistics regarding the morphological features of intracellular hypo-reflective areas after segmentation. The comparisons between CSCR and healthy eyes reveal no significant differences in density (mean), area (mean and SD), perimeter (mean) and circularity (SD) of the segmented areas. However, there are significant differences indicating that hypo-reflective areas in CSCR RPE cells exhibit smaller mean circularity (*P* = 0.008) and solidity (0.0054) factors than healthy RPE cells. These hypo-reflective areas also display greater morphological heterogeneity, as evidenced by the increased dispersion in the solidity (*P* = 0.0013) and perimeter (0.0145) parameters in CSCR eyes (Fig. 8h).

## Discussion

This study has investigated the potential of AO-TFI imaging to visualize RPE with high resolution in patients with CSCR. Our results demonstrate that AO-TFI images reveal RPE changes that correspond to changes detected by standard multimodal imaging, with details enhanced by the addition of cellular resolution. Furthermore, it enables the identification of RPE alterations that were not detected by all clinical imaging modalities.

Specifically, AO-TFI enabled the detailed visualization of hyper-reflective structures observed in en-face IR fundus images. In areas where the neural retina is detached, the accumulation of subretinal fluid masks the RPE, resulting in hypo-reflectivity in IR fundus images, as previously reported^3^. This also leads to blurry AO-TFI images. In cases where IR fundus images showed poorly defined hyper-reflective subretinal deposits under the NSD, corresponding to irregular PED on OCT sections, AO-TFI images revealed a well-defined and circumscribed area with bright components surrounded by a darker edge (Fig. 1). We attribute this enhanced visualization to the interaction between dark-field illumination, inherent to the AO-TFI modality, and the perturbed biological structure. Within this large protuberance, high refractive index components such as melanin or other intracellular components like mitochondria appear towards the periphery. These highly reflective objects can act as biological mirrors capable, deflecting light from dark-field illumination, both inside and outside the eye’s-aperture, thereby generating hyper-reflective signals dependant on the slope. A random structure would induce significant contrast variations, whereas a steep slope, corresponding to the protuberance’s edge, would deflect light from the aperture, resulting in a very low signal.

Under an extra-macular NSD, although RPE cells cannot be accurately visualized, AO-TFI enables the precise visualization of other cells, identified as hyper-reflective foci on OCT cross-sectional images (Fig. 2). These foci, which are not clearly visible on IR images due to their low resolution, were recently analyzed using AO-IRAF imaging by Vienola et al*.*^34^. They suggested that hyper-reflectivity of the foci may result from the accumulation of photoreceptor and RPE fluorophores within macrophages/monocytes. Such hyper-reflective cells were also distinctly visible in resolved CSCR with sequelae, both at the border of the area with ORA thinning and within the area with loss of the RPE line and of the outer retina of the RORA, along with dark dots suggesting the presence of aggregated pigments or melanophages (Fig. 3). With AO-TFI, persistent serous PED forms distinct areas composed of small, dense hyper-reflective dots, surrounded by a larger hyper-reflective edge, suggesting imaging of the elevated photoreceptors (Fig. 4). In contrast, PEDs appear either hypo-reflective on IR images due to attenuation of the signal from the RPE or choroid caused by fluid accumulation^35^, or give no signal at all in areas showing a distinct white halo on en-face enhanced-depth swept-source OCT images^36^.

The most interesting finding is that AO-TFI detected clear RPE abnormalities in 38% of the images captured in regions considered healthy, where no alterations were observed in BAF and IR fundus images and where only minimal changes were noted, if any, in OCT sections. The altered patterns of the RPE, such as hypo-reflective foci surrounded by hyper-reflective structures in AO-TFI (Fig. 7, Figure S4), revealed by AO-TFI but without any detectable signs from other imaging modalities, indicate that some RPE modifications could be more extended than those detected by conventional imaging techniques and may precede the other abnormalities. It remains to be studied whether epitheliopathy could be an underlying component in the pathology, similar to the choroidal vasculopathy that precedes the occurrence of serous detachments in CSCR. While pachychoroid leads to a spectrum of diseases such as pachychoroid pigment epitheliopathy, CSCR, neo-vasculopathy or pachychoroid peripapillary disease^2,5^, it cannot be excluded that RPE structural changes could be an early pathogenic event that could be either independent from the choroidal vasodilation, or even precede the vascular alterations. Longitudinal follow-up should be conducted to evaluate whether the early signs of RPE changes, observed with AO-TFI, can predict areas of oncoming serous detachments, RPE migration or PED, outer retinal degeneration or even choroidal pathogenic events such as neovascularization. This is of importance since to date, the choroid is considered as the primary site of disease, with epitheliopathy being a consequence of pachychoroid^8,47^. In fact, the exact chronology of pathogenic events cannot be ascertained due to the poor sensitivity of our commonly used imaging modalities to analyze the RPE.

By quantitatively analyzing morphological parameters of the normal RPE, as assessed by the other imaging modalities, we detected subtle alterations in the eyes of patients with CSCR. These changes appear as alterations in the shape of intracellular hypo-reflective areas and a greater dispersion in the number of neighboring cells compared to healthy RPE cells. This increased morphological heterogeneity suggests altered RPE monolayer organization in CSCR eyes, and supports the hypothesis that the RPE is affected early. The morphological changes detected in retinal regions that appear healthy in multimodal imaging are indicators of stress on the RPE cell cytoskeleton. The RPE is a monolayer of cuboidal cells that interact apically with the outer segments of photoreceptor cells and basally with Bruch's membrane and the choriocapillaris^48^. Its essential functions depend on its polarization and barrier properties, that rely on the cytoskeleton of individual cells in interaction with junctional complexes. The cytoskeleton made of a highly regulated network of filaments, tubules and associated proteins is controlled by the environment and the extracellular matrix. Any stress condition, whether oxidative, metabolic, mechanic or even aging, affects the cell cytoskeleton which translates into morphological changes of the RPE mosaic organization. This is observed early in hyperglycemic rat models^37^, as well as in human RPE cells submitted to light-induced oxidative stress^38^, and has been extensively characterized during pathological aging in humans^39^. More specifically, in human eyes with AMD, the most notable RPE changes in early-stage disease include the alteration in cell shape with area, solidity, and form factors being the most discriminating descriptors. Interestingly, the reduced BAF in AMD was significantly associated with decreased roundness and solidity^40^, suggesting that the morphological parameters could be linked with other properties of the RPE related to their composition. Using atomic force microscopy, it has been demonstrated that these changes in RPE geometry, reflecting cytoskeleton stress, represent the initial stages of an epithelial-to-mesenchymal transition^41^. Although there are no histological descriptions of the RPE in human patients with CSCR, our results may suggest that the RPE layer is subjected to increased oxidative and/or mechanical and/or metabolic stress, consistent with previous reports of elevated oxidative stress markers in the serum and tears of patients with CSCR^42,43^.

We acknowledge the potential limitations of our study, particularly regarding the use of discretionary areas of interest and the automatic AI-based masking of images. Selecting specific areas for analysis could introduce bias; however, we applied consistent methodologies to all images to mitigate this effect. The quantitative analyses rely on advanced image processing and machine learning algorithms developed by EarlySight, which systematically detect and delineate hypo-reflective areas, mask out low-quality regions, and exclude vessel structures. The use of AI software allows for a more accurate definition of hypo-reflective areas with a non-uniform threshold. Although the software is proprietary, it ensures unbiased and reproducible results by mimicking decisions from multiple human annotators and providing a consistent method across different patient populations, regardless of study objectives. The data generated by the software have been verified by humans to ensure accuracy, offering a reliable method for measuring retinal structures, mitigating biases, and ensuring consistency across different patient populations.

In this first exploratory study, analyzing RPE cells in CSCR eyes, we included patients with varying disease severities, with or without active disease (i.e. subretinal fluid). The advantage of this strategy is the detection of different RPE features with corresponding multimodal images, but its limitation is the low number of eyes with identical abnormalities. We are currently conducting a similar study on larger number of eyes with varying degrees of disease severity, to confirm these preliminary findings. The pigment epithelium, one of the most challenging retinal layers to image, may have been underestimated in the early pathogenesis of CSCR. The results from the present study indicate that extended RPE stress occurs in eyes with CSCR, which might predispose individuals to recurrent serous detachments and /or RPE atrophy or to choroidal neovascularization. Although significant results were observed in this small population, prospective longitudinal studies on larger samples are needed to determine whether RPE morphological characteristics could predict disease progression. AO-TFI images should also be correlated to choroid-specific imaging techniques, such as indocyanine green angiography and OCT angiography, to follow the fate of RPE and choroidal vasculature changes.

In summary, this study demonstrates the value of AO-TFI to better characterize the RPE layer in CSCR. Its ability to visualize subtle RPE changes not easily detectable by other methods highlights its potential for early diagnosis as well as follow-up of CSCR patients. Further research efforts should aim to deepen our knowledge of the roles of the RPE and choroid in CSCR pathogenesis, and to develop predictive markers for disease progression through larger-scale studies and specific imaging techniques.

## Methods

This study (ClinicalTrials.gov: NCT04398394, first registration: 14/05/2020; kofam.ch: SNCTP000003921) was designed and conducted in accordance with the tenets of the Declaration of Helsinki, good clinical practice defined by the International Council for the Harmonization of Technical Requirements for the Registration of Pharmaceuticals for Human Use (ICH) or the ISO 14155, as well as all national legal and regulatory requirements.

The Swiss competent authorities (Swissmedic, #10000613) and the Ethics Committee of the Swiss Federal Department of Health in the Swiss National Clinical trial Portal (Authorization CER-VD n ° 2019-00429) approved the study. Written informed consent was received from all participants prior to inclusion.

### Population

Healthy volunteers over 18 years old and patients with a clinical diagnosis of CSCR were included between the period of August 2020 and April 2022 in the medical retina service of Jules-Gonin Eye Hospital, Lausanne, Switzerland. Only eyes presenting with best corrected visual acuity (BCVA) over 0.6 (decimal), and with a clinical judgment of good central fixation were included.

Exclusion criteria included eyes with significant anterior segment opacities, or in one of the following clinical situations: less than 3 months post- anterior segment surgery (eg cataract), eyes with high myopia (< − 6D), high hypermetropia (> + 5D) and/or significant astigmatism (> + 4D). For patients, exclusion criteria also included eyes with confounding retinopathies or less than 6 months post-surgery of the posterior segment. Not included were also individuals unable to follow the procedures of the study, individuals unable to fix at a target for at least 20 s, persons not tolerant of being in the dark for 30 min, individuals with epilepsy, and individuals with albinism.

### Data and image acquisition

During the screening visit, an ophthalmologist ensured the absence of ophthalmic exclusion criteria. Demographic data collected included age and gender. Refraction was performed and the corresponding spherical equivalents RE (NIDEK RT-6100, NIDEK CO, Japan) was measured along with BCVA and AL (IOL MASTER 700, Carl Zeiss Meditec AG, Germany) measurements for each subject.

All patients underwent anterior segment examination to assess transparency of the cornea and of the lens, as well as retinal imaging using modalities which are part of the standard of care: BAF fundus photography (Optos Daytona P200T, Dunfermline, Scotland) and spectral-domain OCT consisting of 193 B-scans spanning a 20° × 20° area along with the corresponding near IR reflectance images (Spectralis, Heidelberg Engineering, Heidelberg, Germany).

AO-TFI imaging was performed on the day of the screening visit by three trained operators, using the Cellularis® retinal camera (prototype version 1.0, EarlySight SA, Switzerland). For each acquisition, both transscleral beams are used simultaneously to capture 100 raw images. The averaging of this 100-image sequence uses the first image as the reference image to align the others. After high-pass filtering, this processing generates a single high signal-to-noise (SNR) ratio image, few seconds after the acquisition. In each eye, five 5° × 5°high resolution RPE images were captured, four at an eccentricity of 5.4° from the fovea (Z1–Z4) and one centered on the fovea (Z5) (see Supplementary Fig. S1 online). One image (Z6) was captured to include areas of interest at the physician’s discretion.

### Correlative analysis

For each imaged area, the highest-contrast image was visually selected and cropped to remove unaligned edges, then contrast and brightness were automatically adjusted with the dedicated Fiji software tool (ImageJ, version 1.53q).

Stages of CSCR were classified in 3 types of diagnostics: “active CSCR” with neurosensory detachments (NSD) and presence sub-retinal fluid (SRF) on OCT B-scans, “resolved CSCR” without SRF but with retinal alterations on BAF and a history of previous active disease on IR imaging and OCT B-scans, and “healthy contralateral (CL) eyes” without sign of retinal alteration in all clinical images.

The AO-TFI images were then graded depending on changes observed on other en-face imaging modalities including BAF and IR fundus photographs of the same area. The grading was done by two ophthalmologists, with a third intervening in the event of disagreement.

Grade 0 refers to healthy iso-autofluorescent outer retina in BAF fundus images, healthy iso-reflective RPE in IR fundus images and normal RPE cells on AO-TFI images, i.e*.* organized with a honeycomb pattern. Grade 1 refers to NSD, appearing mostly hyperfluorescent in BAF and hypo-reflective in the IR fundus images, and as blurred areas on AO-TFI images. Grade 2 refers to areas where retinal changes are revealed on both BAF, IR and AO-TFI images. Grade 3 involves areas where RPE changes are seen only on IR fundus and AO-TFI images, but not on BAF. Grade 4 involves areas where the retina and RPE are normal on BAF and on IR, but where AO-TFI reveals abnormalities. Any deviation from the healthy RPE pattern was considered abnormal, including: altered mosaic contrast, clusters of hyper-reflective dots and any significant changes in reflectivity.

To correlate AO-TFI images with IR fundus images and OCT B-scans, the most contrasted images selected in each eye were stitched using MosaicJ^44^/TurboReg^45^ to create a montage of the AO-TFI images (see Supplementary Fig. S1 online). Then, a custom semi-automated method was used to correlate the montage of the AO-TFI images with the IR fundus images and the OCT B-scan, using the free software Fiji (ImageJ, version 1.53q). The method consists in two manual steps using Fiji plugins and one automatic step using a custom plugin. The AO-TFI montage was scaled using the equation defining pixel size: a*RE + b*(AL-23.5) + c^33^. Then, the AO-TFI montage was registered on the IR image—OCT field-of-view using Bigwarp^46^. After manual identification of at least 4 landmarks on the vessels in AO-TFI and IR fundus image, the AO-TFI montage is warped after “similarity transformation” consisting in a linear transformation with translation, rotation, and one scale parameter. Two images were exported: the warped AO-TFI montage with the pixel size of the IR fundus image (20 µm/px) and the field of view of “IR fundus-OCT” image, and the high-resolution warped AO-TFI montage (see Supplementary Fig. S2 online). Finally, the custom plugin generated two stacks: one with the mask of the AO-TFI montage on IR-OCT B-scans stack allowing to check the good registration of the low-resolution AO-TFI montage on IR fundus, and one correlating the high-resolution AO-TFI montage with the IR fundus image and the OCT B-scans (see Supplementary Fig. S3 online).

### Quantitative analysis

We used the proprietary software developed by EarlySight SA (beta version of the “Cellularis analytics” software) to perform quantitative image analysis. This consists of the automatic assessment of the quality of images, filtering out areas of the image with low quality, performing tessellation and segmentation of the RPE cells, as well as morphological analyses of the identified shapes.

The quality assessment pipeline uses a machine learning algorithm trained to score AO-TFI images based on their perceived quality, such as blur, lack of focus and SNR ratio. This provides a quality metric (ranging between 0 and 1) for each area of the image, where higher values represent areas with sharp RPE mosaic structures and lower values represent areas with less visible structures. Areas with quality metric values below a certain threshold are masked out and are excluded from quantitative analyses.

The software uses machine learning solutions to provide two types of analyses, namely tessellation and segmentation. The tessellation analysis consists of identifying centers of cells and drawing boundaries between them using the Voronoi algorithm. The Voronoi partitions are then analyzed with common measurements: area (µm^2^), perimeter (µm), equivalent diameter (µm), number of neighboring cells, expressed as mean per and standard deviation (SD) per image—SD measuring the dispersion of these parameters within each image, as well as Voronoi diagram density (cells/mm^2^) and non-masked portion (%). In order to avoid the cells bordering with the masked areas to bias the statistical description of the overall image based on these measurements, the bordering cells are post-filtered and added to the masked areas.

The segmentation analysis consists of identifying the exact contours of the hypo-reflective areas within RPE cells. Once this pixel-level contour description is calculated, morphological analysis of the cells is carried out. This includes various morphometric measurements such as hypo-reflective regions area (µm^2^), perimeter (µm), solidity (ratio of contour area to its convex hull area, a.u.) and circularity (ratio of contour area to its perfect circle area, a.u) expressed as mean and SD per image, hypo-reflective regions density (n/mm^2^), as well as non-masked portion (%).

Only images with non-masked portion over 5% and presenting RPE mosaic were included in the quantitative analysis. We have previously shown that RPE cell densities remain constant in the perifovea of healthy eyes, but decrease with eye elongation and age^33^. To avoid bias in morphometric measurements, parameters from perifoveal images (Z1–Z4) were compared between populations with comparable baseline characteristics (age-matched participants and AL/RE-matched eyes).

### Data analysis

The GraphPad Prism software (version 9.1.2 (226), GraphPad Software, LLC) was used to calculate descriptive statistics and to compare of the means of the variables (age of the participants, eye characteristics, and mean of morphological features per eye) between groups (healthy volunteers versus CSCR) using the unpaired t-test with Welch's correction. *P*-value < 0.05 was considered significant.

### Supplementary Information

Supplementary Legends.

Supplementary Figures.

Supplementary Tables.

Supplementary Movie S1.

Supplementary Movie S2.

Supplementary Movie S3.

Supplementary Movie S4.

Supplementary Movie S5.

Supplementary Movie S6.

Supplementary Movie S7.

Supplementary Movie S8.

## Data Availability

The datasets generated and analyzed during the current study are available from the corresponding author on reasonable request.
