# Supplementary material for: High-resolution adaptive optics-trans-scleral flood illumination (AO-TFI) imaging of retinal pigment epithelium (RPE) in central serous chorioretinopathy (CSCR)
Source: Sci Rep. 2024 Jun 13;14:13689. doi: 10.1038/s41598-024-64524-4 (PMC11176374; doi:10.1038/s41598-024-64524-4)
Supplement: Supplementary file 1 — Supplementary Legends. [file 41598_2024_64524_MOESM1_ESM.pdf]

Description of the supplementary movies

Movie S1. Full stack of 131 "Correlation-fundus-AO-TFI-OCT" images of a healthy contralateral left eye (Male, 40 years). Each image shows the AO-TFI mosaic (center) with the IR fundus cropped to fit the area imaged with Cellularis (left panel) and the corresponding OCT b-scan (right panel). Stack image 117 is shown in the supplemental Figure S3 online.

Movie S2. Full stack of 122 images "Correlation-fundus-AO-TFI-OCT" of a left eye with foveal serous detachment (Female, 43 years). Each image shows the AO-TFI mosaic (center) with the IR fundus cropped to fit the area imaged with Cellularis (left panel) and the corresponding OCT b-scan (right panel). Stack images 61 and 72 stack were used to illustrate the Figure 1.

Movie S3. Full stack of 158 "Correlation-fundus-AO-TFI-OCT" images of a right eye with supero-nasal serous detachment (Male, 40 years). Each image shows the AO-TFI mosaic (center) with the IR fundus cropped to fit the area imaged with Cellularis (left panel) and the corresponding OCT b-scan (right panel). Stack images 96 and 135 were used to illustrate the Figure 2.

Movie S4. Full stack of 143 images "Correlation-fundus-AO-TFI-OCT" of a left eye with retinal atrophy (Male, 47 years). Each image shows the AO-TFI mosaic (center) with the IR fundus cropped to fit the area imaged with Cellularis (left panel) and the corresponding OCT b-scan (right panel). Stack images 32 and 110 were used to illustrate the Figure 3.

Movie S5. Full stack of 150 images "Correlation-fundus-AO-TFI-OCT" of a left eye with pigment epithelial detachment (Male, 46 years). Each image shows the AO-TFI mosaic (center) with the IR fundus cropped to fit the area imaged with Cellularis (left panel) and the corresponding OCT b-scan (right panel). Stack image 127 was used to illustrate the Figure 4.

Movie S6. Full stack of 152 images "Correlation-fundus-AO-TFI-OCT" of a right eye with RPE changes (Male, 41 years). Each image shows the AO-TFI mosaic (center) with the IR fundus cropped to fit the area imaged with Cellularis (left panel) and the corresponding OCT b-scan (right panel). Stack image 112 was used to illustrate RPE changes in Figure 5. Stack images 61 and 95 were used to illustrate healthy RPE in Figure 6.

Movie S7. Full stack of 132 images "Correlation-fundus-AO-TFI-OCT" of a right eye showing RPE contrast changes in AO-TFI (active CSCR, Male, 39 years). Each image shows the AO-TFI mosaic (center) with the IR fundus cropped to fit the area imaged with Cellularis (left panel) and the corresponding OCT b-scan (right panel). Stack images 33 and 112 were used to illustrate the Figure 7.

Movie S8. Full stack of 145 images "Correlation-fundus-AO-TFI-OCT" of a right eye with RPE contrast changes in AO-TFI (resolved CSCR, Female, 47 years). Each image shows the AO-TFI mosaic (center) with the IR fundus cropped to fit the area imaged with Cellularis (left panel) and the corresponding OCT b-scan (right panel). Stack images 14 and 121 were used to illustrate the supplemental Figure S4 online.
