# Supplementary material for: High-resolution adaptive optics-trans-scleral flood illumination (AO-TFI) imaging of retinal pigment epithelium (RPE) in central serous chorioretinopathy (CSCR)
Source: Sci Rep. 2024 Jun 13;14:13689. doi: 10.1038/s41598-024-64524-4 (PMC11176374; doi:10.1038/s41598-024-64524-4)
Supplement: Supplementary file 2 — Supplementary Figures. [file 41598_2024_64524_MOESM2_ESM.pdf]

Figure S1. en face imaging of a healthy contralateral left eye of active CSCR (Male, 40 years).
(a) Blue-iso-fluorescent fundus image. The white square indicates the approximated area imaged with the Cellularis® prototype for AO-TFI. **(b) Iso-reflective infrared fundus.** Squares indicate the areas imaged with AO-TFI: within the fovea (Z5) and the macula at 5.4° eccentricity (Z1, infero-nasal; Z2, infero-temporal; Z3, supero-nasal; Z4, supero-temporal). **(c) Mosaic generated after stitching of the AO-TFI images** using the Fiji plugin MosaicJ. Healthy RPE cells mosaic appears as a honeycomb network of cells with absorbing cores surrounded by bright borders.

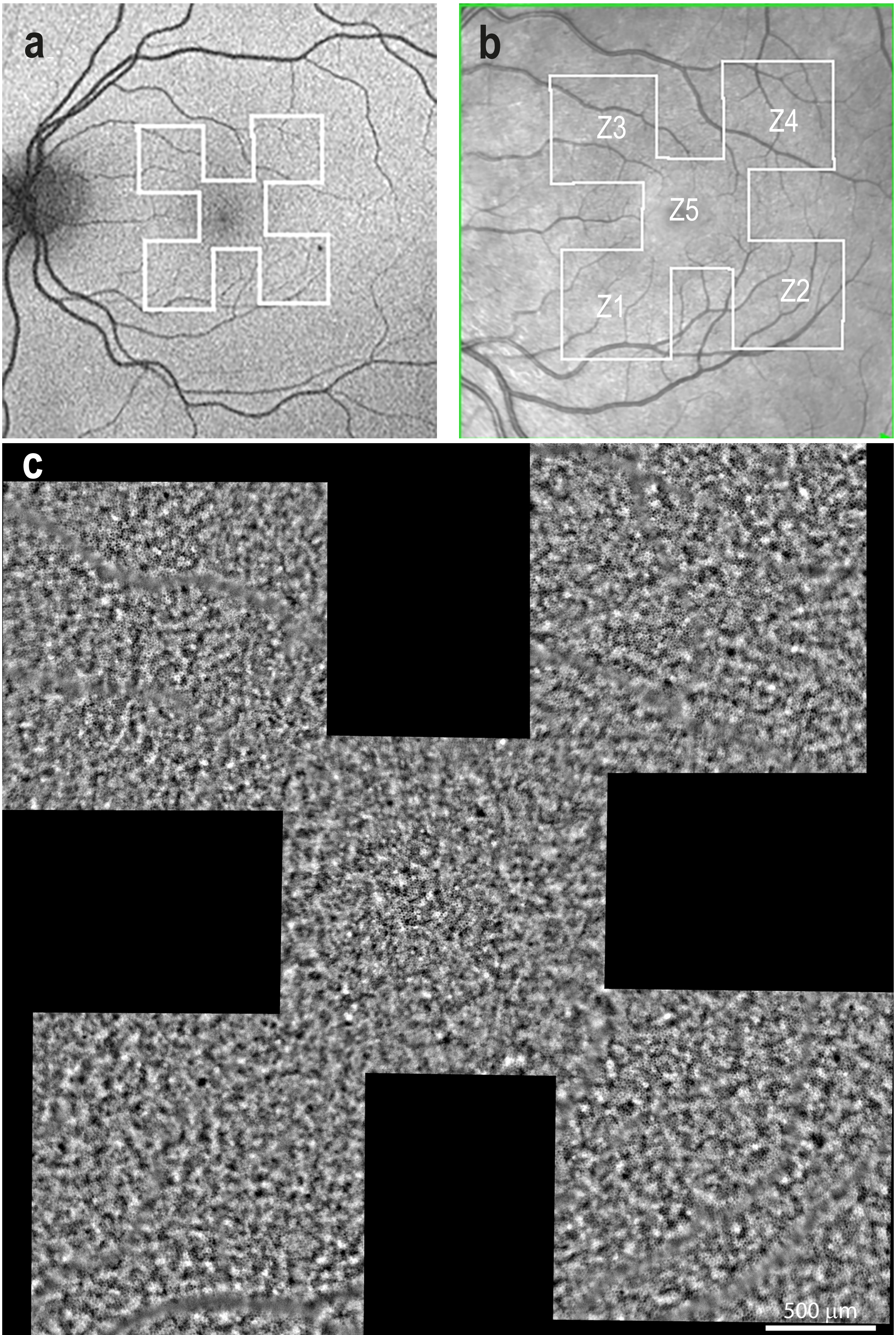

Figure S2. Method to transform the AO-TFI mosaic, using the Fiji plugin Bigwarp.
(a) Landmarks identification on AO-TFI mosaic and infrared (IR) fundus image. Using the "similarity transformation" mode, linear transformation with translation, rotation, and one scale parameter, exportations of two warped images:
(b) the warped AO-TFI mosaic on "IR fundus – OCT B-scan" field-of-view and **(c)** the high-resolution warped AO-TFI mosaic.

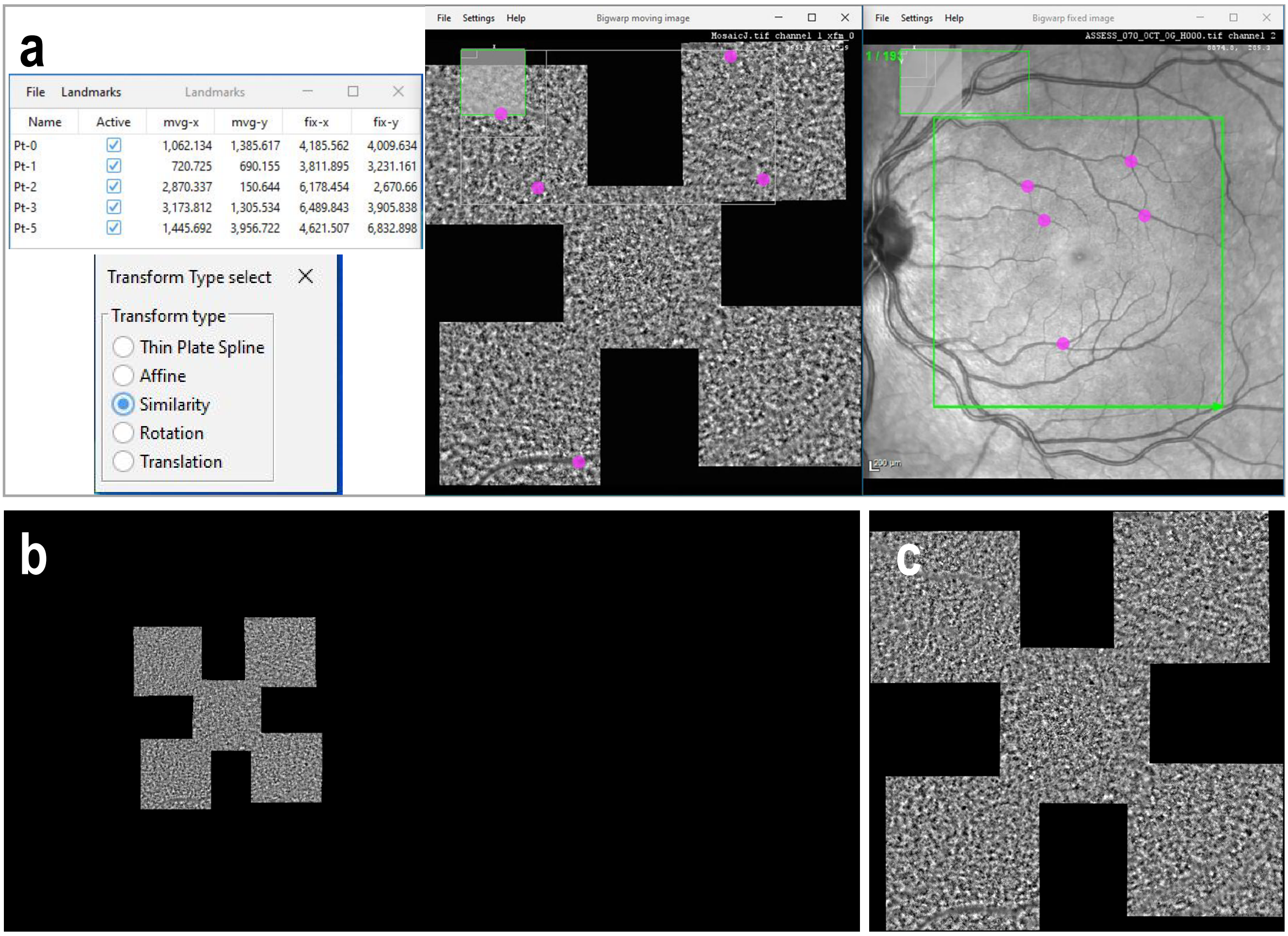

Figure S3. Results of the custom correlation plugin. (a) Registration the mask of the low-resolution AO-TFI mosaic on IR fundus to identify the OCT B-scans corresponding to the AO-TFI mosaic. Example: image #152 over the full "AO-TFI-on-OCT-stack" of 193 images. **(b) Correlation** of the warped AO-TFI mosaic (center) with IR fundus (left panel) and OCT B-scan (right panel) cropped to fit the area imaged with Cellularis. Arrows indicate the exact vessel correlation. The full "Correlation-fundus-AO-TFI-OCT" stack of 131 images is available in Movie S1.

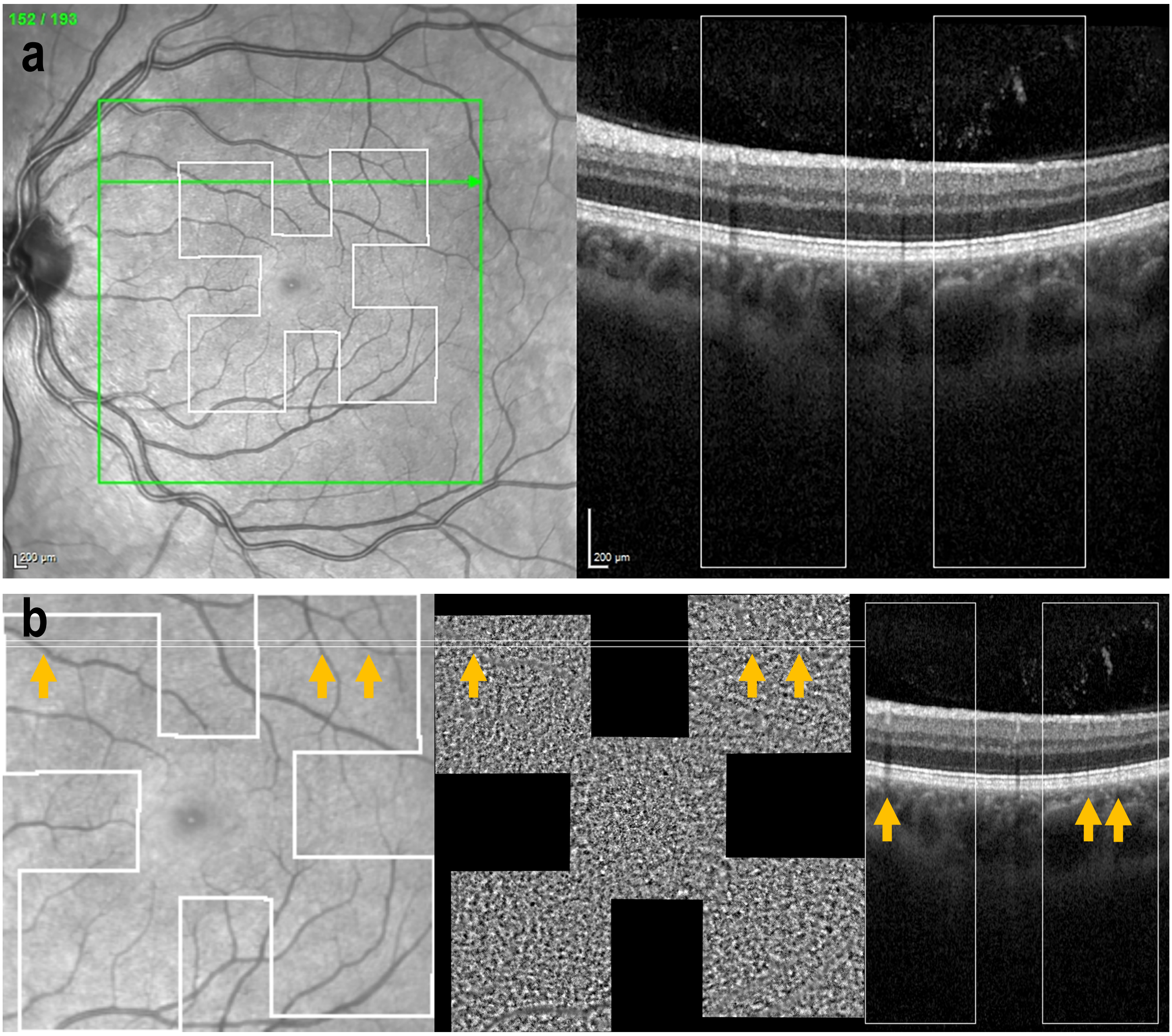

Figure S4. RPE contrast changes on normal BAF and IR fundus areas in resolved CSCR (Female, 47 years).
(a) Infrared fundus image with projection of the AO-TFI mosaic mask (Z5: Grade 2; Z1, Z2, Z3: Grade 3; Z3, Z6: Grade 4). The colored lines indicate the locations of the OCT B-sections shown in panels d (yellow line) and f (blue line).
(b) Blue-autofluorescence fundus image indicating the approximated area imaged with Cellularis® (white square).
(c-d) Correlation of the magnified supero-temporal AO-TFI image (c) with the corresponding OCT B-scan (e).
(e-f) Correlation of the magnified inferior AO-TFI image (e) with the corresponding OCT B-scan (f).
AO-TFI image shows preservation of the RPE cell mosaic and contrast changes, such as hypo-reflective foci surrounded by hyper-reflective structures (insets) corresponding to normal OCT B-scans (d, f).
The complete “IR fundus – AO-TFI – OCT b-scan” correlation is available in Movie S8.

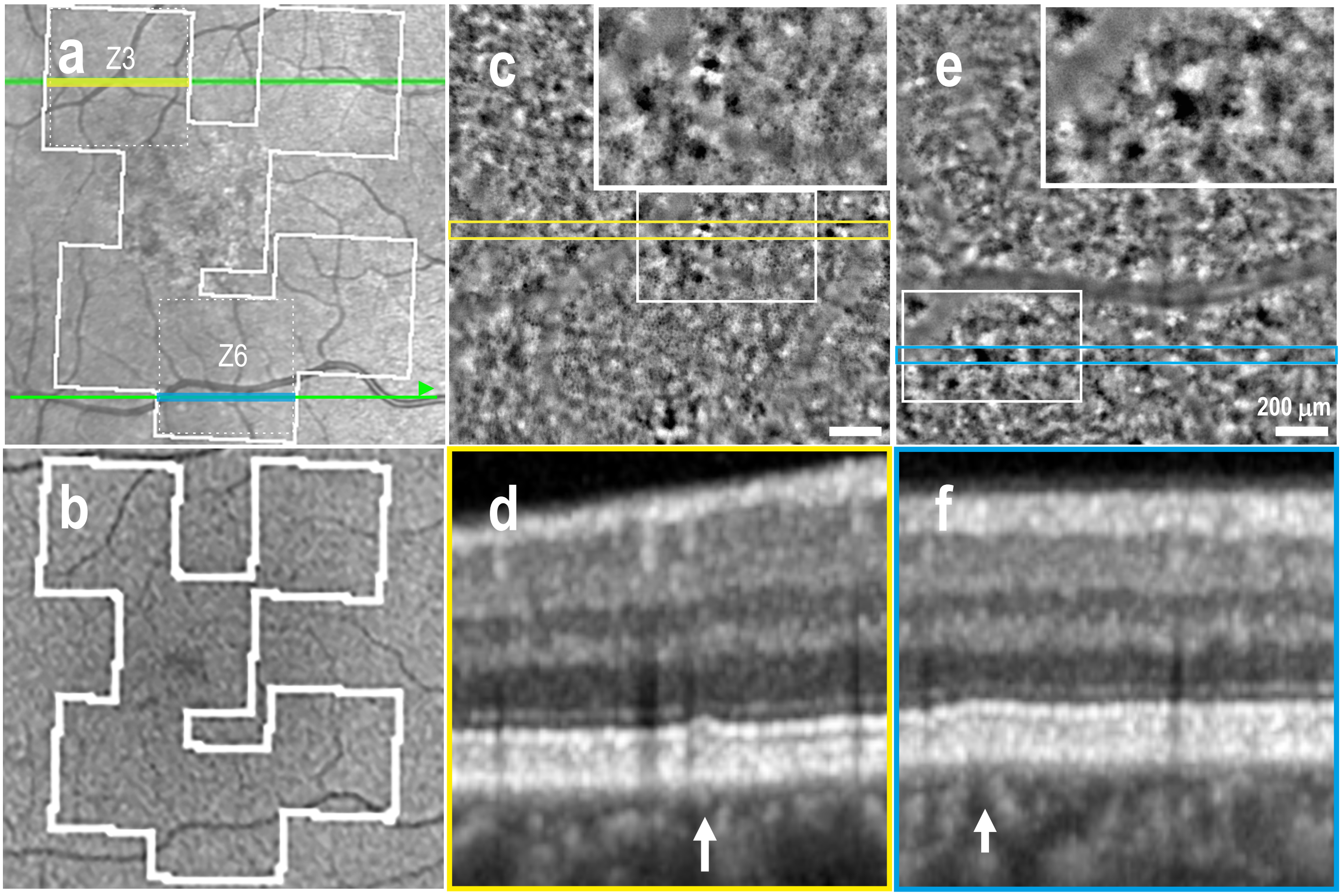

Figure S5. Flow diagram detailing the number of eyes (N), the number of images (n) and baseline (BL) characteristics after **(a)** image selection and **(b)** selection of the eyes included in the quantitative analysis.
After selecting images showing RPE cells organized in mosaics, evaluation of the baseline characteristics of the selected eyes revealed that CSCR eyes had significantly shorter axial lengths, indicating lower myopia compared with healthy eyes. In order to ensure comparability in baseline characteristics, only healthy eyes from participants over 25 years of age with a refractive error (RE) greater than -2 diopters and an axial length (AL) less than 25 mm were included in the analysis.
* Unpaired t test with Welch's correction: healthy controls versus CSCR eyes, excluding healthy CL eyes

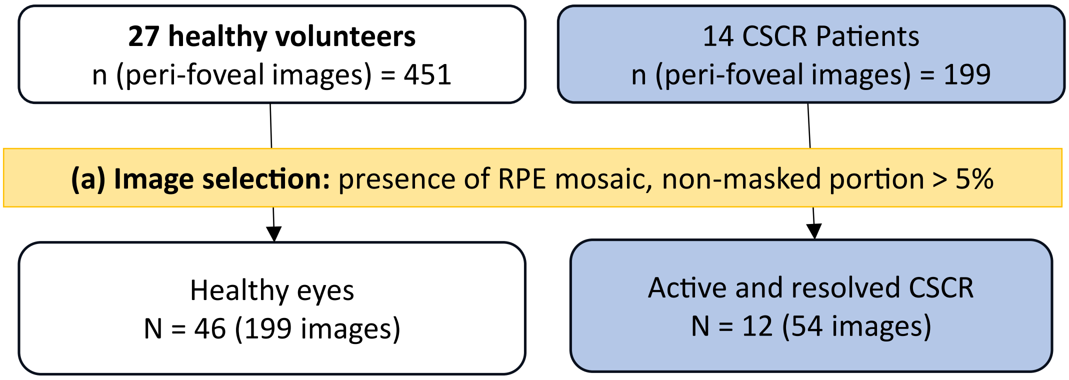

**(a) Nb of participants**	**27**	**Healthy volunteers**			**10**	**CSCR patients**			**t-test***	
Age (years)	Mean	SD	Min.	Max.	Mean	SD	Min.	Max.	P value	
	37.6	13.6	21	70	42.3	5.9	34	54	0.1555	ns
Gender	Female:	9	Male: 18		Female:	2	Male: 8			
**Number of eyes**	**46**	**Healthy eyes**			**12**	**CSCR eyes**			**t-test***	
	Mean	SD	Min.	Max.	Mean	SD	Min.	Max.	P value	
Axial Length (mm)	24.02	0.95	21.88	26.7	23.24	0.78	22.11	24.41	0.0075	**
Refractive Error (Diopters)	-1.06	1.71	-5.75	1.50	0.00	0.45	-1.00	0.88	0.0005	***

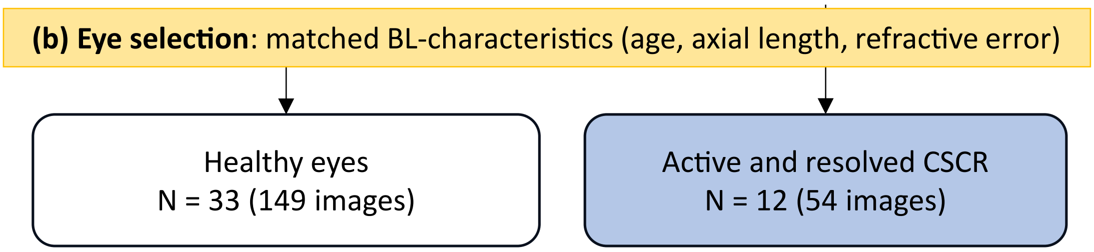

**(b) Nb of participants**	**19**	**Healthy volunteers**			**10**	**CSCR patients**			**t-test***	
Age (years)	Mean	SD	Min.	Max.	Mean	SD	Min.	Max.	P value	
	36.7	12.6	25	64	42.3	5.9	34	54	0.1181	ns
Gender	Female:	7	Male: 12		Female:	2	Male: 8			
**Number of eyes**	**33**	**Healthy eyes**			**12**	**CSCR eyes**			**t-test***	
	Mean	SD	Min.	Max.	Mean	SD	Min.	Max.	P value	
Axial Length (mm)	23.69	0.74	21.88	24.48	23.24	0.78	22.11	24.41	0.0999	ns
Refractive Error (Diopters)	-0.30	0.78	-1.75	1.50	0.00	0.45	-1.00	0.88	0.1254	ns
