# Supplementary material for: High-resolution adaptive optics-trans-scleral flood illumination (AO-TFI) imaging of retinal pigment epithelium (RPE) in central serous chorioretinopathy (CSCR)
Source: Sci Rep. 2024 Jun 13;14:13689. doi: 10.1038/s41598-024-64524-4 (PMC11176374; doi:10.1038/s41598-024-64524-4)
Supplement: Supplementary file 3 — Supplementary Tables. [file 41598_2024_64524_MOESM3_ESM.pdf]

Supplementary Table S1. Descriptive statistics of the participant and eye baseline characteristics. The calculated pixel size (µm) = a*RE + b*(AL-23.5) + 0.74, where a =-0.0022 and b=0.0031 comes from the linear regression based on a simulation of the optical system of the Cellularis prototype [31]. BCVA, Best-Corrected Visual Acuity; Hg, Mercury; IOP, Intraocular Pressure; RE, Refractive Error

N= 24 eyes from 14 patients with central serous chorioretinopathy (CSCR; 3 Females, 11 Males)

N= 46 eyes from 27 healthy volunteers (9 Females, 18 Males)

Group		Age (years)	Axial Length (mm)	Spherical Equivalent RE (Diopters)	Calculated pixel size (µm)	BCVA (Logmar)	IOP (mm Hg)
CSCR	Mean	43.4	23.26	-0.05	0.73	-0.10	12.63
	SD	5.3	0.68	0.59	0.02	0.07	2.22
	Min.	34.0	22.11	-1.50	0.70	-0.20	9.00
	Max.	54.0	24.57	1.25	0.77	0.10	18.00
Healthy	Mean	37.6	24.02	-1.06	0.76	-0.12	14.74
	SD	13.6	0.95	1.71	0.03	0.06	3.29
	Min.	21.0	21.88	-5.75	0.69	-0.20	8.00
	Max.	70.0	26.70	1.50	0.85	0.00	23.00

Supplementary Table S2. Grading of the AO-TFI images per stages of CSCR (Active, Resolved, healthy contralateral, CL). Out-of-focus foveal images were not graded (n/a).

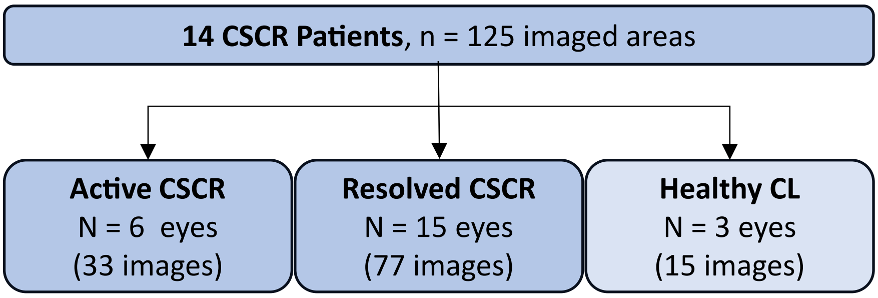

Grade	Active CSCR			Resolved CSCR			Healthy CL			Total		
	n(images)		N(eyes)	n(images)		N(eyes)	n(images)		N(eyes)	n(images)		N(eyes)
**n/a**	1	3%	1	4	5%	4	0	0%	0	5	4%	5
**1**	7	21%	5							7	6%	5
**2**	12	36%	5	27	35%	13	0	0%	0	39	31%	18
**3**	4	12%	3	18	23%	13	0	0%	0	22	18%	16
**4**	4	12%	2	10	13%	6	6	40%	2	20	16%	10
**0**	5	15%	3	18	23%	8	9	60%	2	32	26%	13

Supplementary Table S3. Statistics on morphological characteristics of Voronoi diagrams after tessellation.For each parameter, means ± standard deviation (SD) correspond to the mean of the means per eye in each group. * Unpaired t test with Welch's correction

	**Healthy volunteers**		**CSCR patients**			
**Parameters measured**	n =	33 eyes	n =	12 eyes	t-test*	
**after tesselation**	Mean	SD	Mean	SD	P value	
**Non-masked portion (%)**	22.00	11.87	22.46	13.05	0.641	ns
**Density (mm)**	6189	662	6372	490.6	0.3266	ns
**Area mean (μm^2)**	165.60	17.14	160.10	11.76	0.2357	ns
**Area SD (μm^2)**	49.87	5.18	50.02	3.55	0.9124	ns
**Perimeter mean (μm)**	50.48	2.51	49.88	1.76	0.3786	ns
**Perimeter SD (μm)**	6.98	0.41	7.21	0.37	0.0846	ns
**Number of neighbors mean**	5.93	0.03	5.92	0.03	0.376	ns
**Number of neighbors SD**	1.01	0.02	1.03	0.02	0.001	***
**Diameter mean (μm)**	14.34	0.75	14.11	0.50	0.2379	ns
**Diameter SD (μm)**	2.16	0.14	2.20	0.10	0.3231	ns

Supplementary Table S4. Statistics on morphological characteristics of hypo-reflective areas after segmentation. For each parameter, means ± standard deviation (SD) correspond to the mean of the means per eye in each group. * Unpaired t test with Welch's correction

	**Healthy volunteers**		**CSCR patients**			
**Parameters measured**	n =	33 eyes	n =	12 eyes	t-test*	
**after segmentation**	Mean	SD	Mean	SD	P value	
**Non-masked portion (%)**	28.46	13.23	29.74	14.97	0.5577	ns
**Density (μm)**	6146	619	6312	473	0.3467	ns
**Area mean (μm^2)**	20.81	2.59	20.34	2.10	0.541	ns
**Area SD (μm^2)**	16.59	1.72	17.71	2.09	0.114	ns
**Perimeter mean (μm)**	20.15	1.30	19.98	1.06	0.6581	ns
**Perimeter SD (μm)**	9.98	0.66	10.69	0.80	0.0145	*
**Circularity mean (a.u.)**	0.677	0.009	0.670	0.005	0.0008	***
**Circularity SD (a.u.)**	0.131	0.004	0.132	0.001	0.2486	ns
**Solidity mean (a.u.)**	0.849	0.007	0.844	0.004	0.0054	**
**Solidity SD (a.u.)**	0.107	0.003	0.109	0.002	0.0013	**
